# Spatiotemporal Mapping of the Contracting Gravid Uterus of the Rabbit Shows Contrary Changes With Increasing Gestation and Dosage With Oxytocin

**DOI:** 10.3389/fendo.2019.00802

**Published:** 2019-11-21

**Authors:** Corrin M. Hulls, Roger G. Lentle, Wei-Hang Chua, Philip Suisted, Quinten M. King, Joana A. B. Chagas, John P. Chambers, Lauren Stewart

**Affiliations:** ^1^Medical Physiology Research Unit, School of Health Sciences, College of Health, Massey University, Palmerston North, New Zealand; ^2^Division of Obstetrics and Gynaecology, Palmerston North Hospital, Palmerston North, New Zealand; ^3^Division of Urology, Palmerston North Hospital, Palmerston North, New Zealand; ^4^School of Veterinary Science, Massey University, Palmerston North, New Zealand

**Keywords:** motility, spatiotemporal mapping, uterus, oxytocin, salbutamol, gestation, birth

## Abstract

Spontaneous and oxytocin induced contractile activity was quantified in the bicornuate uteri of pregnant rabbits maintained *in situ*, using data from two- and uni- dimensional video spatiotemporal maps (VSTM) of linear and area strain rate and compared statistically. Spontaneous contractions occurred over a range of frequencies between 0.1 and 10 cpm, in gravid animals at 18–21 and at 28 days of gestation, and propagated both radially and longitudinally over the uterine wall overlying each fetus. Patches of contractions were randomly distributed over the entire surface of the cornua and were pleomorphic in shape. No spatial coordination was evident between longitudinal and circular muscle layers nor temporal coordination that could indicate the activity of a localized pacemaker. The density and duration of contractions decreased, and their frequency increased with the length of gestation in the non-laboring uterus. Increasing intravenous doses of oxytocin had no effect on the mean frequencies, or the mean durations of contractions in rabbits of 18–21 days gestation, but caused frequencies to decrease and durations to increase in rabbits of 28 days gestation, from greater spatial and temporal clustering of individual contractions. This was accompanied by an increase in the distance of propagation, the mean size of the patches of contraction, the area of the largest patch of contraction and the overall density of patches. Together these results suggest that progressive smooth muscle hypertrophy and displacement with increasing gestation is accompanied by a decrease in smooth muscle connectivity causing an increase in wall compliance and that oxytocin restores connectivity and decreases compliance, promoting volumetric expulsion rather than direct propulsion of the fetus by peristalsis. The latter effects were reversed by the β2 adrenergic receptor agonist salbutamol thus reducing area of contraction, and the duration and distance of propagation.

## Introduction

The principal mechanical functions of the mammalian uterus are to act as a repository in which fetal growth can take place and to provide a means by which the fetus can be expelled once fully developed. The former is achieved by amelioration of the tone in the walls of the uterus and hence their compliance i.e., the ease with which the uterine cavity may be dilated. Hence cavity volume is increased, in human subjects from around 50 ml (1) to over 4,000 ml (2), so as to accommodate the growing fetus without undue elevation of intra-luminal pressure (3). However, little is known of the changes in contractile dynamics that underlie this “accommodative” modulation of tone and, furthermore, there is conflicting evidence regarding the spatiotemporal disposition of uterine contractions.

A body of indirect evidence regarding the disposition and timing of individual contractions comes from the qualitative analyses of changes in associated electrical potential in multi-electrodes (4). The bulk of this work suggests that the propagation of electrical phenomena associated with uterine contractions i.e., localized bursts of activity, is chaotic rather than ordered, with locally varying patterns with re-entry evident in some species (4, 5). A further a body of work suggests that uterine contractions do not progress circumferentially along the long axis of the uterine cavity. Hence for example uterine contractions (6) and associated electrical activity (7) are relatively reduced around the site of placentation (8). Again, whilst work indicates that nature and timing of uterine contractions can be modified by hormonal and other stimuli (9), we can find no direct evidence that these stimuli facilitate the spatiotemporal organization of contractions across the full thickness of the uterine muscle to engender peristaltic progression. Qualitative direct studies of human uterine contraction using two dimensional electro-hysterographic mapping similarly show that the propagating front of electrical burst activity is irregular (10) and that uterine contractions vary in frequency and strength between proximal and distal regions and between adjacent sites (6, 11).

Together such findings support a hypothesis that the movement of uterine contents in the appropriate direction could be produced solely by a change in intrauterine pressure i.e., an overall increase in the tone of the wall so as to reduce lumen volume, in both rabbits and in human subjects (12, 13). Further, evidence suggests that such action could result solely or partly from local mechano-transduction (14). Thus, strips of uterine muscle are sensitive to stretch (15) and a mechano-transductive response occurs when a bolus of Tyrodes solution is injected into the uterine cavity. The latter comprises an initial “early stretch” response when luminal pressure rises above baseline pressure and a subsequent period when rhythmic contractions decrease in amplitude and intra-uterine pressure declines over a period of 20 min (16). Similarly a number of experiments have shown that hydrostatic (17) and tensional (18) forces can evoke similar contractions in the pregnant uterus. Hence, impairment in the magnitude of mechanoreceptor evoked micro-contractions may act to reduce intrauterine pressure and secure accommodation in a similar manner to that in capacious structures such as the resting urinary bladder (19, 20) and gastric fundus (21). Again hormonal (22) and other stimuli (23) may act to alter the threshold of such resetting so as to increase intrauterine pressure and engender fetal expulsion. Hence for example oxytocin is reported to increase the frequency and intensity of contractions in the ostrogenized uterus via a variety of pathways (24).

A number of other studies have suggested that uterine contractions are spatiotemporally organized. An early paper showed orderly progression of electrical activity in the uterine cornua of pregnant ewes (25). Again, whilst recent multi-electrode studies showed little evidence of tightly ordered proximal to distal propagation of individual uterine contractions, the broad overall direction of the development of propagating electrophysiological bursts was reported to be from proximal to distal along the long axis of the uterus (26–29). Recent ultrasonographic studies based on cross sectional data have also suggested that the inner, predominantly circular, layer of uterine muscle next to the endometrium i.e., the *stratum subvasculare* may become spatiotemporally organized to form “peristaltic like” contractions (30). More recently, concerted histological and electrophysiological work in the isolated uterus of the rat has indicated the presence of myometrial/placental pace-making zones that are closely associated with the site of placentation, prompting a hypothesis that “spatial organization of these areas likely promotes coordinated delivery of fetuses in a polytocus uterus” (31).

*Sensu strictu* peristalsis comprises the integrated action of steadily propagating bands of longitudinal and circular muscle contraction to produce a propagating luminal constriction that bears directly against, and imparts impulsion to, the contents of the lumen (32, 33). In the small intestine such integrative action is aided by the anatomical and physiological separation of the longitudinal and circularly orientated smooth muscle (34). There is a similar anatomical demarcation of the longitudinal and circular muscle in the uterine wall of certain species including that of species with a bicornuate uterus such as the rabbit (35). However, the orientations of the long axes of the component myocytes in the wall of the human uterus appear to be less sharply demarcated, although recent work indicates there are higher concentrations of circularly orientated fibers near the cavity and around the tubal openings, and higher concentrations of longitudinally orientated fibers near the serosa (36). Other work indicates that the wall of the human uterus consists of localized masses of smooth muscle (37) whose long axes are extensively intertwined (36), an anatomy which led one worker to hypothesize that this structure and the manner of its interconnection by interstitial tissue provide for local mechanical function (38).

Were uterine contractions organized to form peristalsis in order to directly propel the uterine content then we would expect that their direction of propagation would vary with function. Hence, the human uterus is reported to exhibit intermittent contraction (39) during menstruation to void cellular debris (40), during the proliferative phase of the menstrual cycle to secure retrograde transport of spermatozoa (41) and during early pregnancy to secure the proper placement of the conceptus in the uterine cavity (42). Currently we can find no definitive evidence that these events are organized to propagate in the appropriate direction.

Conversely, if the uterus were to act primarily on a basis of mechanotransduction and the movement of the contents to be consequent on changes in volume or intrauterine pressure, then the direction of their movement could be determined by concomitant reciprocal variation in the level of occlusion of the proximal i.e., tubal, or the distal i.e., cervical openings of the cavity rather than by change in the spatial organization and sequence of contraction. This would obviate any need for changes in direction of contraction via neural or myogenic means.

In the current work we use various video spatiotemporal mapping (VSTM) techniques to directly quantify and statistically evaluate the temporal and spatial form, area, and coordination of spontaneous contractions in the wall of the pregnant uterus of the rabbit maintained *in situ* prior to and at term and after dosage with increasing quantities of oxytocin and after subsequent dosage with salbutamol. This, with a view to comparing the spatial and temporal characteristics of contractions during the accommodative and expulsive phases of uterine action during pregnancy with those obtained from multi-electrode electro-physiological and other studies. Further, to determine whether in the latter phase the spatial pattern of contractions are consistent with direct propulsion of contents by peristalsis or by expulsion from the resetting of uterine tone.

## Materials and Methods

All the experimental procedures were approved by the Massey University Animal Ethics Committee (MUAEC approval number 17/100), and complied with the New Zealand Code of Practice for the Care and Use of Animals for Scientific Purposes.

### Anesthesia

Each rabbit was maintained on 100% oxygen in a clear acrylic induction chamber for 5 min prior to administration of the anesthetic. Halothane (4%) was then given in 100% oxygen until the animal lost its righting reflex and surgical anesthesia induced with alfaxalone IV (2–3 mg/kg) via a 20 G catheter in the cephalic vein. The animal was then intubated with a 3.5 mm cuffed endotracheal tube and anesthesia subsequently maintained by a combination of halothane (EtHal 0.8–1.2%) in oxygen and intravenous alfaxalone maintained at 0.1–0.2 mg/kg/min.

Arterial blood gases were monitored by a 22 G catheter and mechanical ventilation used to maintain normocapnia. Peak systolic blood pressure was maintained above 60 mmHg with intravenous noradrenaline (0.5–1.5 μg/kg/min) when necessary, and body temperature was maintained at 38–39°C via forced-air warming.

### Procedure

Eight pregnant New Zealand white rabbits were obtained from a commercial breeder and maintained on commercial feed, which was available *ad libitum* with water until immediately prior to the procedure.

**Figure 1:**
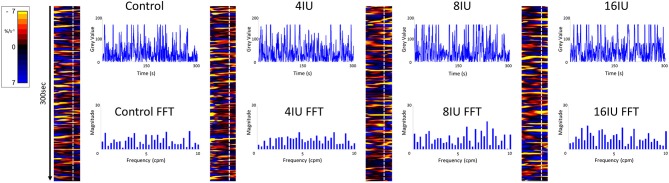
Unidimensional plots of temporal and positional variation in area strain rate, vertical transects and their fast Fourier transforms before and following IV administration of increasing doses of oxytocin from a rabbit uterus at day 18 of gestation.

Following administration of anesthetic (see above) the animal was placed in a supine position and the abdomen was opened with a vertical ventral paramedian incision sited lateral to the line of the breast tissue. The anterior surface of the left cornu of the uterus was exposed and lightly dusted with carbon black. The animal was then rotated to the left into a semi-prone position so that the left cornu of the uterus, and contained fetuses, could prolapse laterally through the incision into an organ bath perfused with oxygenated (95% O_2_, 5% CO_2_) Earle-Hepes buffered saline (HS) of pH 7.35, comprising 124.0 mM NaCl, 5.4 mM KCl, 0.8 mM MgSO_4_, 1.0 mM NaH_2_PO_4_, 14.3 mM NaHCO_3_, 10.0 mM Hepes, 1.8 mM CaCl_2_, and 5.0 mM glucose maintained at 37°C and continuously recirculated at a flow rate of 160 ml/min (Figure 1).

The carbon-dusted anterior surface of the left cornu was adjusted to face the video camera (Supplementary Figure 1). Myoelectrical activity in uterine wall was recorded with two multi-stranded stainless-steel wire electrodes (see below). Note that it was not possible to insert electrodes into the area of the uterus undergoing VSTM as the presence of the wire interfered with the video image and hence the subsequent analysis.

Image sequences were recorded on a video camera mounted above the organ bath to capture the three most distal fetuses in the left cornu of the uterus and saved as uncompressed AVI video files for off-line processing. Hence the motility pattern over the surface of each cornu was subsequently evaluated by cross-correlation techniques in one or two dimensions using the techniques described below.

An intravenous line was inserted through which requisite doses of the pharmaceutical agent oxytocin was given (Phoenix. Vetpharm (NZ) Ltd). Oxytocin is a known specific activator of uterine contractility at term (43). Salbutamol (GlaxoSmithKline (NZ) Ltd) is known to inhibit contractions in the gravid uterus (44). Salbutamol was added directly to the organ bath superperfusate to give a bath concentration of 174 nmol/L. The latter route was chosen to minimize the generation of any systemic e.g., cardiovascular effects that could have a confounding effect on uterine motility.

At the conclusion of the recordings the anesthetized animal was euthanized with an IV bolus of pentobarbitone (125 mg/Kg) (National Veterinary Supplies Ltd. Auckland, New Zealand). The crown rump lengths of the fetuses from the eight pregnant rabbits were subsequently determined post-mortem and used to determine their gestational age (45) and hence the duration of the pregnancy. This enabled the preparations to be categorized on a basis of their gestation.

The temporal correlations of the various parameters derived by spatiotemporal mapping were subsequently correlated with those of the electrophysiological recordings. The variations in the various quantified temporal and spatial parameters of uterine contractions were subsequently compared according to gestational age so as to identify any significant cross correlation.

Video spatiotemporal mapping (VSTM) allows the local movement of the distinctive visual features (in this case formed by the carbon particles) between successive frames to be quantified by the displacements of reference points on a grid of equally spaced points within a rectangular region of interest (ROI). Linear strain rates can be recorded either along the longitudinal or the radial dimensions of the uterine cornu. Conventionally, strain rates have a negative value when the distance between a given pair of markers is decreasing i.e., the tissue is contracting, and a positive value when the distance between markers is lengthening i.e., the tissue is expanding. Thus L-type spatiotemporal (ST) maps i.e., unidimensional plots of the variation in linear strain rate at all points along an appropriately curved longitudinal line of interest (LOI) and along a straight radial LOI (x-axis) over time (y-axis), were generated from the video recordings (Figure 1). The longitudinal LOIs were positioned along the central axis of the anterior surface of the cornu and the radial LOIs orthogonal to this i.e., vertically across the anterior surface overlying the mid-point of the fetus.

The speed and direction (radial or longitudinal) of propagation of a burst of contractions, or of its component individual contractions, across the surface of the uterus can be directly determined from these unidimensional maps. Hence, the (acute) angle formed between the front of a propagating contraction and the horizontal axis is inversely proportional to its speed of propagation, a shallow angle indicating a faster speed. The direction of movement of the propagating front is indicated by the direction of the acute angle i.e., left to right or right to left. The frequencies of the burst type contractions can be determined from the points of intersection of successive bursts on a vertical line on the ST map in the time dimension. Further, the profile of frequencies between 1 and 10 cycles per minute (cpm) of all contractile events including burst contractions could be assessed from Fast Fourier transforms taken along the same vertical transects of the L-type ST maps over a period of 300 s, derived in Microsoft Excel. The Fast Fourier transforms could then be compared between the two gestational age groups and between the three doses of oxytocin within each group. The amplitudes and durations of the individual and burst contractions can be determined from vertical transects and similarly compared.

Area strain rates (ASR) at each reference point on the ellipses can be determined from the local displacement rates of markers using the same technique that was described in a previous paper (19). Briefly, ASR are expressed as the percentage change in muscle area per unit time, i.e., % s^−1^. Like linear strain rates, ASRs have a negative value when area is decreasing and a positive value when area is increasing. Unidimensional plots of the variation in ASR over time (A-type plots) can be plotted in the same manner as L-type maps. Alternatively two-dimensional plots of variation in ASR can be overlaid onto real time video frames to produce two dimensional A-type plots. The sensitivity of the ASR mapping method is well-established. Hence, for example, ASR maps derived from myocardial MRI have been shown to provide better discrimination between normal and ischemic zones than other indices of strain (46).

A sequence of uni-dimensional (A-type or L-type) and two-dimensional (A-type) ST maps, were prepared. The latter were superimposed on corresponding video images of the uterine cornu to enable the patterns of motility on its surface to be directly visualized. The area of superimposition was limited by a user-specified, standardized ellipsoidal masks with their longitudinal axes located on and aligned with the longitudinal axis of the cornua and their radial axis position across the midline of the fetal outline. Each data set was taken from an ellipse that occupied 45% of the anterior uterine surface. This proportion was chosen in order to exclude all sites that were close to the edge of the organ profile in which artifacts from rotational movement of the organ and parallax could occur (Figure 1). Hence the 45% area of the 18–21 day gestation group (110 mm^2^) was significantly smaller than that of the 28 day gestation group (695 mm^2^). Thus, comparisons of data between the two gestational ages could be confounded by differences in total area. Hence a further data set was taken in which the area of the ellipse on the 28 day group identical to that on the 18 day group i.e., 110 mm^2^ with the longitudinal axis of the ellipse similarly located on the longitudinal axis of the cornu so as to standardize for area. The resulting ASRs were color-coded such that rapidly contracting areas appeared yellow (–ve ASR), more slowly contracting areas appeared red and expanding areas appeared blue (+ve ASR).

The two dimensional parameters of groups of propagating patches of contraction (PPCs) within the ellipse of A-type ST maps of controls and following the various treatments were each determined from 300 video frames taken at 1 s intervals over a 5 min period. Each original video image was imported into ArcGIS (v10.4 1999-2015 Esri Inc). In this analysis component pixels in which the strain rate was below −4% s ^−1^, were classified as contracting and colored yellow. Correspondingly, component pixels where strain was zero, or greater than −4% s ^−1^ i.e., stretched, were classified as not contracting and colored blue.

## Electrophysiology

Myoelectrical activity in the uterine wall was recorded synchronously with VSTM by a pair of stranded stainless-steel wire electrodes (Part no. AS632, Cooner Wire Company, Chatsworth, California, USA). The bared tips (1–2 mm long) of the electrodes were implanted into the muscle layer by puncturing the outer serosa with a sterile 23 G needle which also served to insert the wire electrode. The exposed tips of the electrodes were hooked to ensure they remained secure in the muscle. The bipolar electrodes were placed strategically in a location on the gravid uterus that was close to the site of VSTM. Each pair of electrodes were positioned ~4–5 mm apart. A grounding needle electrode was inserted subcutaneously on a bony face of the tibia of each subject. The electrodes were connected via shielded cables to a bio amplifier (Animal Bio amp ML136, AD Instruments, Dunedin, New Zealand) and Powerlab data acquisition system (Power-lab 8/35, AD Instruments). Raw myoelectric data was recorded using LabChart 8 Pro v8.1.13 at a rate of 1 kbyte per second and stored on a PC for future analysis. This was filtered with a band-pass digital filter set between 0.2 and 40 Hz so as to distinguish contractile activity from gross movement artifacts and line noise.

## Further Data Processing and Statistics

Mean durations of uterine contractions were derived from pixel counts of consecutive events on 300 s vertical transects of area type i.e., A-type maps. Mean frequencies were calculated from fast Fourier transforms of the same vertical transects.

We adapted parameters that were originally developed in the FRAGSTATS software suite (47) to describe the spatial structure of patches of vegetation in landscapes to quantify the changes in shape and size of uterine contractions during their development and involution. Hence successive classified raster plots were exported from ArcGIS as GeoTIFF's, for processing by FRAGSTAT, i.e., in order to determine (47):-

1. Patch density (%PLAND). The total areas of all patch contractions occurring in a given A-type ST elliptical map as a percentage of the total area of the ellipse,
2. Largest Patch Index (LPI). The mean area of the largest patch contraction as a percentage of the total area of the ellipse,
3. Number of Patches (NP). The mean number of patches within the ellipses,
4. MPS. Mean patch size in mm^2^.

The values for the various parameters were subsequently compared by one way ANOVA or by repeated measures ANOVA, where relevant, in IBM® SPSS® Statistics (Version 25) to determine the significance of differences between gestational age and the effects of administration of oxytocin and salbutamol. Unless otherwise stated, all results are presented as the mean ± SE of the mean observation for each animal.

## Results

Spontaneous local uterine contractile activity was successfully recorded in four rabbits at mid gestation (18–21 days) and four at late gestation (28 days) and after increasing IV doses of oxytocin and subsequent dosage with salbutamol at various sites across the left uterine cornu.

### Mid Gestation (18–21 Days)

#### Unidimensional Plots

Unidimensional plots of variation in linear (see later) and area strain rate (Figure 1) each showed short-lived contractions that occurred over a range of frequencies from 0.5 and 10 cpm and propagated rapidly over short distances in the radial and in the longitudinal plane. Consequently, they were angled slightly to the left or right of the ST map. There was no tendency for the direction of propagation of an individual contraction to change (Figure 1).

**Figure 2:**
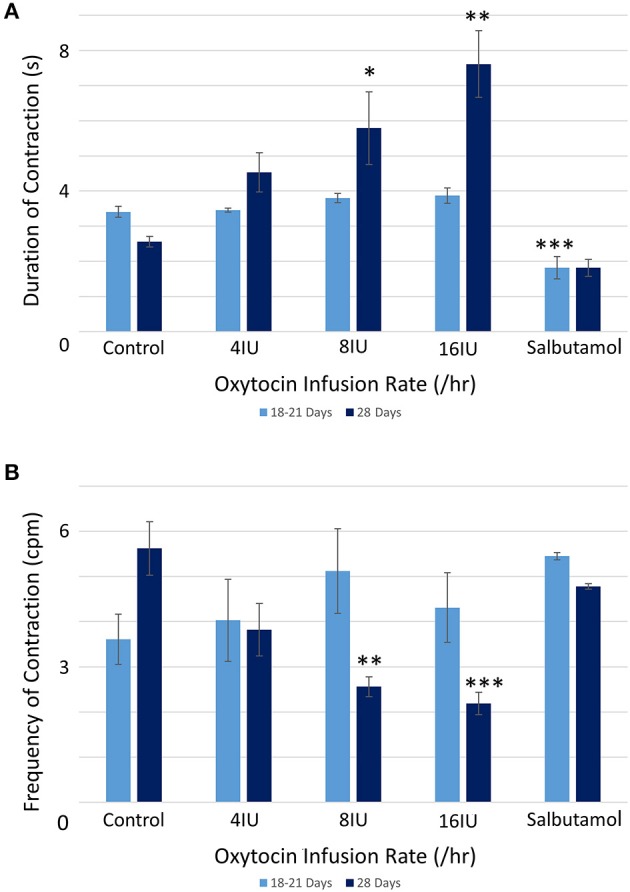
Effects of gestational growth, oxytocin and salbutamol on durations **(A)** and frequencies **(B)** of uterine contractions in the gravid rabbit at 18–21 days and at 28 days on parameters derived from unidimensional VSTM. Differences from control on repeated measures ANOVA following dosage with IV oxytocin; *p < 0.05, **p < 0.01, ***p < 0.001.

There were no significant differences, on repeated measures ANOVA, in the durations or the frequencies of contractions or their direction of propagation (Figure 2 and Table 1), following the IV administration of increasing doses oxytocin.

**Table 1:** Quantitative data derived from vertical transects of ST maps of variation in strain rate from contraction in the gravid uterus of the rabbit.

**Duration of contraction (s)**	**Control**	**4 IU/h**	**8 IU/h**	**16 IU/h**	**Salbutamol**
**OXYTOCIN INFUSION RATE**					
Day 18–21 of Gestation	3.4 ± 0.16	3.46 ± 0.06	3.80 ± 0.13	3.87 ± 0.22	1.82 ± 0.32^***^
Day 28 of Gestation	2.56 ± 0.15^##^	4.53 ± 0.56	5.79 ± 1.03^*^	7.61 ± 0.95^**^	1.82 ± 0.24
**Frequency of Contractions (CPM)**					
Day 18–21 of Gestation	3.61 ± 0.56	4.03 ± 0.91	5.12 ± 0.94	4.31 ± 0.77	5.45 ± 0.08
Day 28 of Gestation	5.62 ± 0.59^#^	3.82 ± 0.58	2.56 ± 0.22^**^	2.19 ± 0.25^***^	4.78 ± 0.06

*Mean durations of contractions were derived from pixel counts of consecutive contractions on 300 s vertical transects of unidimensional A-maps. Mean frequencies were calculated from fast Fourier transforms of the same vertical transects*.

*Differences between controls at different gestations on one way ANOVA*;

# *p < 0.05*,

## *p < 0.01, N = 4*.

*Differences between corresponding treatment and control on one way ANOVA*;

* *p < 0.05*,

** *p < 0.01*,

*** *p < 0.001, N = 4*.

#### Two Dimensional Plots

Two dimensional plots of variation in area strain rate showed spontaneous short- lived contractile activity occurred in pleomorphic patches at all sites on the uterine cornu in all rabbits of 18–21 days gestation (Supplementary Figure 2). These patches increased in size by peripheral growth and by aggregation with smaller contractions and decreased in size by the reverse process. The sites of such intermittent contraction were randomly distributed across the entire radial surface of the cornua overlying and around each fetus.

**Figure 3:**
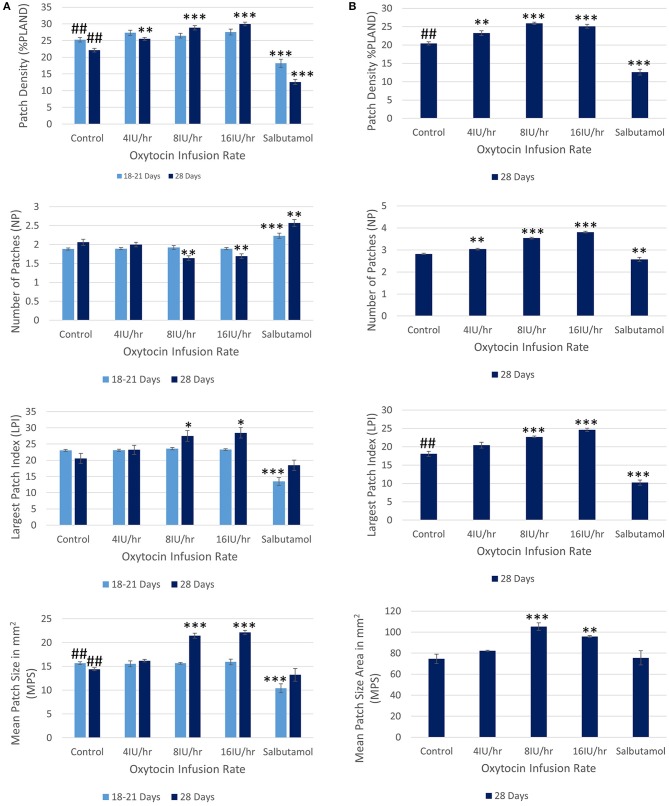
Effects of gestational growth, oxytocin and salbutamol on parameters derived from two dimensional VSTMs of the gravid rabbit uterus using **(A)** an ellipse of 110 mm^2^ at 18–21 days and at 28 days and **(B)** an ellipse of vs. 695 mm^2^ at 28 days only. Differences from control on repeated measures ANOVA following dosage with IV oxytocin; *p < 0.05, **p < 0.01, ***p < 0.001. Differences between controls on one way ANOVA; ^*##*^*p* < 0.02.

Following treatment with increasing IV doses of oxytocin there were no significant increases, on repeated measures ANOVA, either in the densities of contractions within the 45% ellipse i.e., contraction density, or in mean number of patches, the largest patch index or the mean patch area (Figure 3A and Table 2A).

**Table 2A:** Variation of Indices of contractile activity within ellipses of A-type maps with gestation, oxytocin, and ellipse size.

**Day 18–21 of Gestation fragStat metrics**	**Control**	**4 IU/h**	**8 IU/h**	**16 IU/h**	**Salbutamol**
**OXYTOCIN INFUSION RATE**					
Patch density (% Landscape of Mask)	25.27 ± 0.67##	27.33 ± 0.80	26.47 ± 0.70	27.54 ± 0.88	18.21 ± 1.21^***^
Number of Patches!!break (NP)	1.88 ± 0.03	1.89 ± 0.03	1.92 ± 0.05	1.89 ± 0.03	2.23 ± 0.07^***^
Largest Patch Index!!break (LPI)	23.02 ± 0.33	23.09 ± 0.36	23.59 ± 0.39	23.29 ± 0.26	13.48 ± 1.22^***^
Mean Patch Size Area in mm^2^ (Area MN)	15.67 ± 0.28##	15.51 ± 0.62	15.65 ± 0.18	15.93 ± 0.60	10.41 ± 0.94^***^
**Day 28 of Gestation!!break fragStat metrics**					
Patch density (% Landscape of Mask)	22.10 ± 0.55##	25.51 ± 0.43^**^	28.83 ± 0.66^***^	29.96 ± 0.57^***^	12.59 ± 0.79^***^
Number of Patches!!break (NP)	2.06 ± 0.08	2.00 ± 0.06	1.64 ± 0.06^**^	1.69 ± 0.06^**^	2.57 ± 0.09^**^
Largest Patch Index!!break (LPI)	20.57 ± 1.56	23.24 ± 1.42	27.50 ± 1.66^*^	28.46 ± 1.59^*^	18.46 ± 1.59
Mean Patch Size Area in mm^2^ (Area MN)	14.39 ± 0.32##	16.18 ± 0.27^*^	21.43 ± 0.53^***^	22.12 ± 0.47^***^	± 1.3

*Comparison on a basis of standard ellipse size (110 mm^2^)*.

### Late Gestation (28 Days)

#### Unidimensional Plots

**Figure 4:**
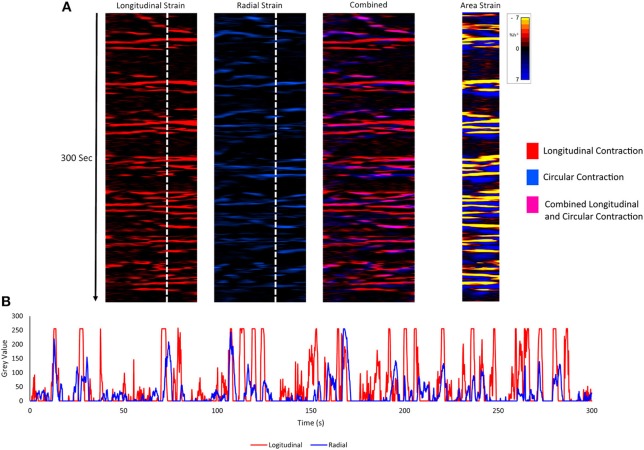
Component unidimensional plots of radial and longitudinal strain rate and area strain rate in the cornua of a rabbit uterus at 28 days of gestation. Consecutive columns in **(A)** are concurrent. The plots of linear strain rate in the longitudinal (red) (1st column) and radial (blue) (2nd column) direction, the overlay of the two (with longitudinal shown as pink) (3rd column) and the concurrent map of area strain rate plot (4th column) show no tendency to form consistent sequences of contraction. Superimposition of plots from vertical transects of first and second columns **(B)** demonstrates the lack of synergy between contractions in radially and longitudinally orientated smooth muscle.

**Figure 5:**
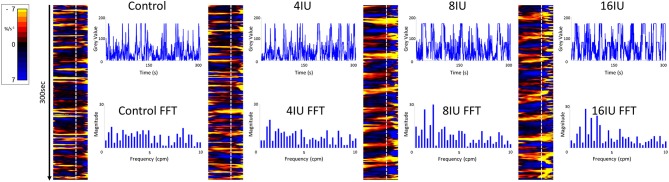
Unidimensional plots of variation in area strain rate, their vertical transects and fast Fourier transforms of contractile activity in a uterine cornu of a gravid rabbit of 28 days gestation, before and following increasing IV doses (4, 8, and 16 U) of oxytocin. Note the increase in duration and decrease in predominant frequencies at the higher doses of oxytocin.

Unidimensional plots of variation in linear (Figure 4) and area (Figure 5) strain rate showed no evidence of spontaneous emergence in late gestation of consecutive, radially disposed, bands of circular and longitudinal contractions, such as are seen in peristalsis, either in the radial or the longitudinal transects of plots of variation in area strain rate. Similarly, there was no evidence of temporal or spatial coordination after increasing doses of oxytocin (Figure 5). Thus, localized longitudinal and circular components of contractions continued to occur irregularly, and on occasion concurrently, with no evidence of temporal or regional (Figure 5) organization.

At 28 days gestation the mean frequency of contractions was significantly increased, and the durations significantly reduced, on one way ANOVA compared with those at 18–21 days gestation (Figures 3A,B and Table 1).

Conversely, increasing IV doses of oxytocin (from 8 to 16 IU) caused local contractions to become grouped into broader, more regular, composite bands of significantly longer duration and lower frequency on one way ANOVA (Figures 3–5 and Table 1). The changes in mean frequency were also reflected in the fast Fourier transforms of the vertical transects of the unidimensional maps of variation in area strain rate, by an increase in the lower frequencies around 10 cpm (Figure 5). There was also an increasing tendency for the composite burst contractions to propagate proximally rather than distally and to propagate across the entire length of the unidimensional plot (Figure 5).

**Figure 6:**
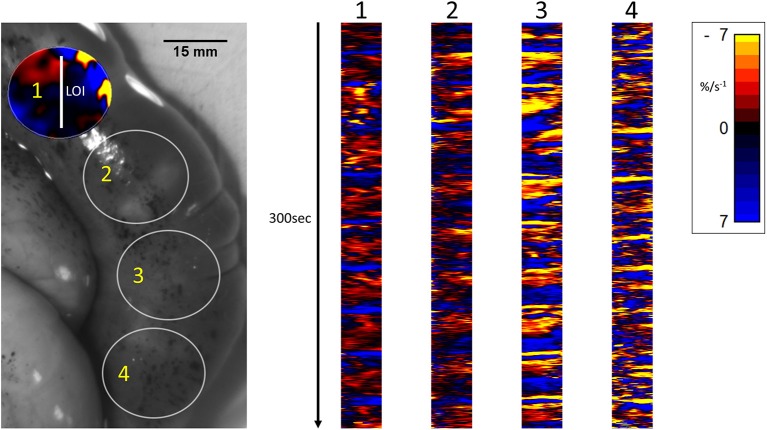
Temporal sequence of unidimensional maps showing variation in area strain rate from contractile activity in adjacent fetuses in a rabbit uterine cornua of day 28 gestation, following IV administration of IV dosage with 16IU oxytocin. The circles **(Right)** represent the sites of VSTM sampling for L Maps **(Left)** site 1 overlies the distal pole of the first fetus in the cornua, site 2 the interval between the fetuses (distended with displaced amniotic fluid), site 3 the proximal pole of the next fetus and site 4 the distal pole of that fetus.

Unidimensional area strain rate plots taken with LOIs traversing two successive fetuses and spanning the region between the two (Figure 6), showed that on occasion, composite contractions propagated from the wall that overlay one fetus onto the wall overlying the neighboring fetus. However, the bulk of the data showed no consistent correlation of the activities in the two sites (Figure 6).

#### Two Dimensional Plots

**Figure 7:**
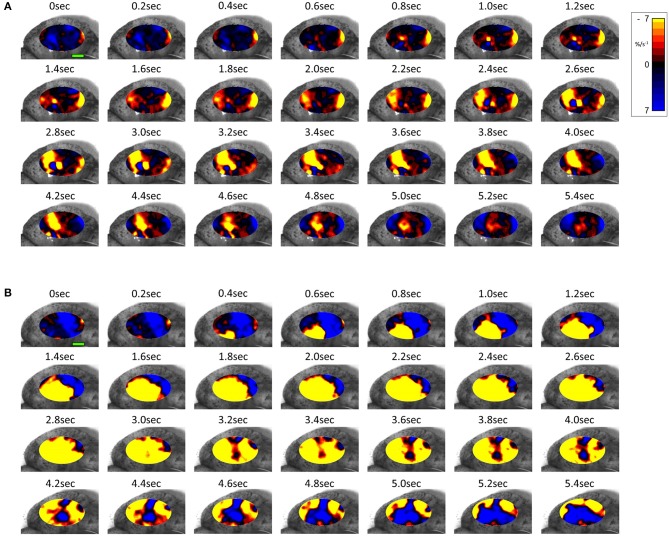
Temporal sequence of two-dimensional maps showing changes in area strain rate from spontaneous contractions in a representative rabbit uterus at 28 days gestation before **(A)** and after dosage with oxytocin **(B)** overlaid onto the anterior surface of the uterus. Decrease in size from active contraction shown as red (high) and yellow (medium) levels of negative strain rate with stasis or relaxation shown in blue. Green Scale bar at time 0 s of each contractile sequence represents 10 mm.

The same spontaneous short- lived contractile activity occurred in pleomorphic patches at all sites on the uterine cornu in all rabbits of 28 days gestation (Figure 7A and Supplementary Videos 1, 2) as did at 18–21 days gestation (Supplementary Figure 2). When the patterns of spontaneous contractile activity in the controls at 28 days gestation were compared with those at 18–21 days gestation, using an ellipsoid mask of identical size (110 mm^2^) there were no significant differences either in the number of patches or areas of largest patches (LPI) (Figure 3A) (Table 2A). Again, with the smaller mask, both the densities of contraction (%PLAND) and mean patch size (MPS) were significantly lower on ANOVA at 28 days gestation than at 18–21 days gestation (Figure 3A and Table 2A). However, the values for MPS were significantly greater when contraction data obtained with the (larger) 695 mm^2^ ellipse taken at 28 days gestation was compared with the data from the (smaller) 110 mm^2^ ellipse taken at 18–21 days (Figure 3B and Table 2B), indicating that patch size increased with the uterine enlargement, but there was not a significant decrease in contraction density (%PLAND).

**Table 2B:** Comparison on a basis of percentage surface area of cornua (45% of width i.e., 110 mm^2^ at v18–21 day vs. 695 mm^2^ at 28 days).

**FragStat Metrics**	**Control**	**4 IU/h**	**8 IU/h**	**16 IU/h**	**Salbutamol**
**OXYTOCIN INFUSION RATE**					
% Landscape of Mask!!break (PLAND) Δ	20.45 ± 0.44^##^	23.23 ± 0.64^**^	25.90 ± 0.35^***^	25.09 ± 0.54^***^	12.59 ± 0.79^***^
Number of Patches!!break (NP) Δ	2.82 ± 0.03	3.04 ± 0.04^**^	3.54 ± 0.04^***^	3.81 ± 0.04^**^	2.57 ± 0.09^**^
Largest Patch Index!!break (LPI) ΔΔ	18.07 ± 0.69	20.42 ± 0.78	22.67 ± 0.23^***^	24.59 ± 0.43^***^	10.23 ± 0.70^***^
Mean Patch Size Area!!break in mm^2^ ΔΔ (Area MN)	74.50 ± 4.41^##^	82.22 ± 0.59	105.18 ± 3.58^***^	95.77 ± 0.87^**^	75.50 ± 6.81

*Data was obtained from A-type ST maps of strain rate. Each parameter value was determined from 300 video frames (one every second over a period of 5 min) N = 4 animals in all cases*.

*Differences from control on repeated measures ANOVA following dosage with IV oxytocin*;

* *p < 0.05*,

** *p < 0.01*,

*** *p < 0.001*.

*Differences between controls on one way ANOVA*;

## *p < 0.02*.

*Differences on doubly repeated measures ANOVA comparing responses to IV oxytocin at day 18–21 with those at day 28 as the significance of interaction term between gestation and oxytocin responses; Δp < 0.001, ΔΔp < 0.005*.

Whilst none of these parameters changed significantly after treatment with increasing doses of oxytocin at 18–21 days gestation, they increased significantly following dosage at 28 days gestation (Figure 3B and Supplementary Video 2 and Table 2B). Hence, there was a significant increase in the contraction density (%PLAND) on repeated measures ANOVA within the 695 mm^2^ ellipses in rabbits of 28 days gestation (*p* < 0.05, df 3.9, *f* = 18.46) (Figure 4 and Table 2B). Similarly, there were significant increases on repeated measures ANOVA in the largest patch index and in MPS (Figure 3B and Table 2B). Similar results were obtained with data from the smaller mask size (110 mm^2^) (Figure 3A and Table 2A).

#### Effects of Salbutamol After Oxytocin

Addition of salbutamol directly to the organ bath superperfusate, to a concentration of 174 nmol/L, following treatment with increasing doses of oxytocin at 20 days caused the contractions to fragment into their short-lived, component, individual micro-contractions (Figures 2, 3 and Supplementary Figure 3 and Tables 1, 2) with corresponding reduction in their duration and distance of propagation despite the lack of effect of oxytocin. Hence there were significant reductions on ANOVA in duration [*F*_(4, 15)_ = 17.77, *p* = 0.004] %PLAND [*F*_(4, 15)_ = 19.72, *p* = 0.0003], largest patch index (LPI) [*F*_(4, 15)_ = 49.14, *p* = 0.0005], and the number of patches [*F*_(4, 15)_ = 11.22, *p* = 0.0005] and compared to those after dosage with oxytocin.

Addition of salbutamol directly to the organ bath superperfusate, to a concentration of 174 nmol/L, following treatment with increasing doses of oxytocin at 28 days caused the relatively prolonged contractions of lower frequency and longer duration that formed after dosage with oxytocin to fragment into their short-lived, component, individual micro-contractions (Figures 2, 3 and Supplementary Figure 3 and Tables 1, 2) with corresponding reductions in their duration and distance of propagation. Hence there were significant reductions on ANOVA in duration [*F*_(4, 15)_ = 22.28, *p* = 0.002] and %PLAND [*F*_(4, 15)_ = 130.02, *p* = 0.0005] and number of patches [*F*_(4, 15)_ = 27.37, *p* = 0.0005] and compared to those after dosage with oxytocin.

Hence salbutamol had similar effects after dosage with oxytocin regardless of gestational age and the lack of any significant effects of oxytocin at 20 days gestation.

### Electrophysiology

The electrophysiological recordings taken at sites adjacent to those of VSTM (a total of four rabbits) had overall frequencies of burst type events that were of a similar order of magnitude to those of micro-contractions with a similar relative increase following dosage with IV oxytocin (Supplementary Figure 4). Hence the mean frequencies during control periods (N = 4) was 1.72 (SE 0.86), that following 4 IU of oxytocin was 2.39 (SE 1.20), that following 8 IU was 2.75 (SE 1.37), and that following 16 IU was 3.48 (SE 1.74). Likewise the frequencies in these recordings decreased 0.51 (SE 0.29) following addition of salbutamol .

## Discussion

This is the first study to use one and two-dimensional VSTM to directly quantify the location and timing of uterine contractile activity at different stages of gestation and in response to oxytocin and salbutamol. The results provide new insights into gestational changes in the mechanics of myometrial contraction as well as providing reciprocal illumination regarding the results of existing electrophysiological studies (27).

Hence, our work shows that ongoing, spontaneous, pleomorphic, localized patches of contraction occurred on an ongoing basis at sites that were distributed across the entire surface of the uterus throughout the middle and later stages of pregnancy. Further, that their overall frequency increased and duration decreased with increase in length of gestation whilst their frequency decreased and their duration increased following the administration of oxytocin. Whilst this finding fits in with prior electrophysiological work (5, 27, 48) and in this sense are not novel, it is important to confirm these prior findings as the validity of electrophysiological evidence has recently been called into question. Hence it was hypothesized that the results of multi-electrode studies in the gastric body and antrum were an epiphenomenon that resulted from the relative movements of electrodes relative to the tissue during its contraction (49, 50). In the case of the gastric antrum, the criticism was in part refuted by concurrent spatiotemporal mapping and electrophysiological recording in the anterior surface of the antrum when myogenic contraction was pharmacologically inhibited (51), but similar work has not until now been carried out with regard to uterine contractile activity.

The ongoing nature of contractions found in this study also fits in with prior studies showing that ongoing oscillations in amniotic pressure that were associated with uterine contractions continued throughout pregnancy (52). Similarly, they fit in with work measuring localized changes in intra-myogenic pressure at various sites in the uterine wall (53) and with the results of a number of electrophysiological studies using multiple electrodes (27).

The pattern of aggregation of adjacent patches of contraction and their subsequent decay by the reverse process (Figure 7 and Supplementary Figure 2 and Supplementary Videos 1, 2) suggests that their disposition is governed primarily by local myogenic rather than concerted neurogenic activity. This statistically supported conclusion, based on the direct quantitative spatiotemporal data, fits in with reported electro-physiological events i.e., irregular, sometimes re-entrant, patterns of propagation of excitation (27).

**Figure 8:**
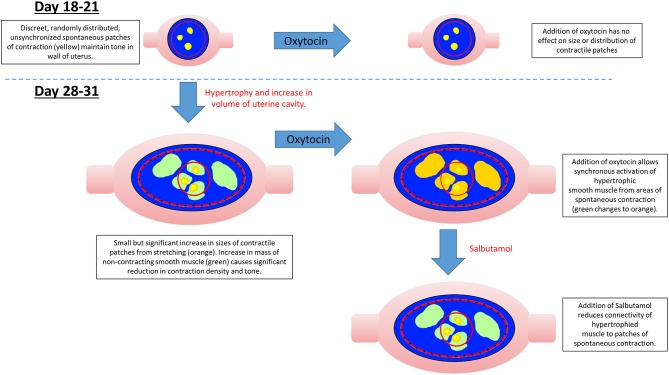
Possible mechanism for variation in the distribution, size, and number of spontaneous uterine contractions in the gravid rabbit uterus with gestation, oxytocin, and salbutamol.

The significant overall decrease in the density of patch contractions and their duration, and the increase in the frequencies of contractions with the length of gestation, together suggest that the concomitant increase in uterine size and cavity volume from smooth muscle hypertrophy (54) results in a decrease in connectivity between adjacent myocytes (Figure 8). This in turn causes the compliance of the uterine wall and its mechano-sensitivity to progressively decrease, allowing the uterine cavity to accommodate the growing fetus (Figure 8). Whilst it is possible that such accommodation could result from extrinsic neurogenic signaling, our results suggest that it is myogenic in origin (see below). This fits in with the findings that the capacity of the uterus to accommodate the growing fetus is preserved in uterine transplants in which extrinsic neural connections have necessarily been severed (55), and the fact that no functional intrinsic neutral network has yet been identified in the uterine wall.

Given the reported increase in densities of oxytocin receptors in late pregnancy (43), the tendency at late gestation for component patches of contraction to be larger, of longer duration and to propagate over greater distances after dosage with oxytocin, fit in with the electrophysiological findings that electrical connectivity between myocytes increases in later pregnancy (56) and the progressive recruitment of action potentials to form more sustained, faster moving, composite bursts of contractions (5, 57–59) (Supplementary Video 2).

The finding that contractions that originate at sites on the uterine wall which overlie one fetus may on occasion propagate to neighboring fetuses, indicates the extent of such increased connectivity and mitigates against a hypothesis of a single localized peri-placental origin of excitation (48) i.e., pacemaker in this species.

Together the findings regarding the spatiotemporal distribution of uterine contractions after the administration of increasing doses of oxytocin render it unlikely that the reorganization of contractile activity during late gestation could induce concerted direct distal-ward propulsion of a contained fetus and thus could be classified as peristaltic. Hence, uterine contractile activity in late gestation continued to consist of pleomorphic patches that were randomly distributed across the surface of the uterus around and overlying each fetus, did not form distally progressing bands, and did not comprise synchronous separate longitudinal and circumferential patches of contraction such as have been reported in peristalsis (60) (Figure 6). Further, the overall trend of direction of propagation of patches was from distal to proximal, rather than from proximal to distal. It is therefore more likely that late gestational reorganization progressively reduces the mean compliance of the uterine walls and thus promotes expulsion by volumetric reduction. The lack of any consistent site of origin of contractions, notably in regions adjacent to the placenta (31) suggests that under our experimental conditions, where the uterus was maintained *in situ*, there was no pacemaker-induced coordination of contractile activity. The trend of distal to proximal progression of patches of contraction possibly results from uterine smooth muscle being drawn from the proximal pole of each fetus toward the distal pole.

The reorganization of smaller individual patches of uterine contraction into larger, more numerous, pleomorphic propagating patches of lower frequency and longer duration following dosage with oxytocin at 28 weeks gestation is similar to the changes that occur in the isolated urinary bladder following administration of cholinergic agents and promotes expulsion of urine (19, 61). Hence in both cases it appears that the changes in the patterns of contraction accompany a change in function from accommodation to expulsion and may involve a local resetting of myogenic connectivity, although that of the bladder occurs more promptly than that of the uterus.

The effect of salbutamol in reducing the duration and overall distance of propagation of composite contractions and inducing their fragmentation into component individual contractions regardless of the length of gestation, fits in with its reported pharmacological action in reducing the excitability of myocytes via the generation of cAMP (62, 63) and the connectivity of myocytic tight junctions (64). Whilst it is possible that it could act to reduce the excitability of intermediary cells such as interstitial cell of Cajal-like cells, their reported electro-physiological characteristics do not fit in with such a role (65). The overall effects of salbutamol in reducing the overall incidence of contractions and hence overall uterine tone are in line with the reported effects on general contraction (66) and cavity pressure (67) in the intact uterus of the rat.

Apart from providing a greater understanding of uterine function, the reciprocal changes in the frequency duration, area and density of uterine contractions that were found to occur with increasing gestation, and following dosage with oxytocin at 28 days, may provide a useful means for identifying the switching of uterine function from accommodation to expulsion, although further work is needed to confirm that similar changes occur in human subjects. Again, the demonstration that the VSTM methodology is able to directly quantify changes in the development and disposition of uterine contractions in the rabbit preparation following administration of pharmaceuticals lays the ground for statistically based assays of other agents that influence myometrial contractility at various stages of uterine function.

## Data Availability Statement

All datasets generated for this study are included in the article/Supplementary Material.

## Ethics Statement

The animal study was reviewed and approved by Massey University Animal Ethics Committee.

## Author Contributions

CH, RL, QK, and PS: surgery. CH: data analysis. W-HC: electrophysiology. JC, JPC, and LS: veterinary care and anesthesia. All contributed to writing of the manuscript.

### Conflict of Interest

The authors declare that the research was conducted in the absence of any commercial or financial relationships that could be construed as a potential conflict of interest.

## Supplementary Material

Supplementary Figure 1 Experimental setup for recording VSTM in the gravid uterus of the anesthetized rabbit. The anterior surface of the prolapsing uterus is positioned in the organ bath with its anterior surface to the right and filmed by an appropriately positioned video camera. The elliptical mask of the VSTM covered 45% of its anterior surface. The electrophysiological electrodes were positioned just outside of the filmed area. PC, personal computer; BA, bioamplifier; CM, video camera; SP, syringe pump; PP, peristaltic pump; HS, reservoir of Earle –Hepes solution.

Supplementary Figure 2 Temporal sequence of two-dimensional maps showing variation in area strain from spontaneous contractions in a representative rabbit uterus at 20 days gestation before **(A)** and after **(B)** dosage with 16 U of oxytocin. Decrease in size from active contraction shown as red (high) and yellow (medium) levels of negative strain rate with stasis or relaxation shown in blue. Green scale bar at time 0 s in each contractile sequence represents 10 mm.

Supplementary Figure 3 Temporal sequence of unidimensional maps showing variation in area strain rate from component contractions after commencement of continuing maximal dosage with oxytocin a rabbit uterus at 18–21 and 28 days gestation and subsequent dosage with salbutamol (174 nmol/L).

Supplementary Figure 4 Temporal sequences of myoelectric activity in the wall of the gravid uterus of the rabbit taken concurrently with VSTM. Representative tracings (300 s duration) of myoelectrical activity in the wall of the gravid rabbit uterus (28 days) of resting activity (Control), and that stimulated by IV oxytocin at various doses (4, 8, 16 IU) and reduction of activity with Salbutamol. All traces were filtered (band pass 0.2–40 Hz) for potential gross movement artifacts and line noise.

Supplementary Video 1 Spontaneous contractile activity in the rabbit uterus at day 28 of gestation.

Supplementary Video 2 Spontaneous contractile activity following dosage of 16IU oxytocin in the rabbit uterus at day 28 of gestation.
