# Supplementary figures and images for: Spatiotemporal Mapping of the Contracting Gravid Uterus of the Rabbit Shows Contrary Changes With Increasing Gestation and Dosage With Oxytocin
Source: Front Endocrinol (Lausanne). 2019 Nov 21;10:802. doi: 10.3389/fendo.2019.00802 (PMC6882407; doi:10.3389/fendo.2019.00802)

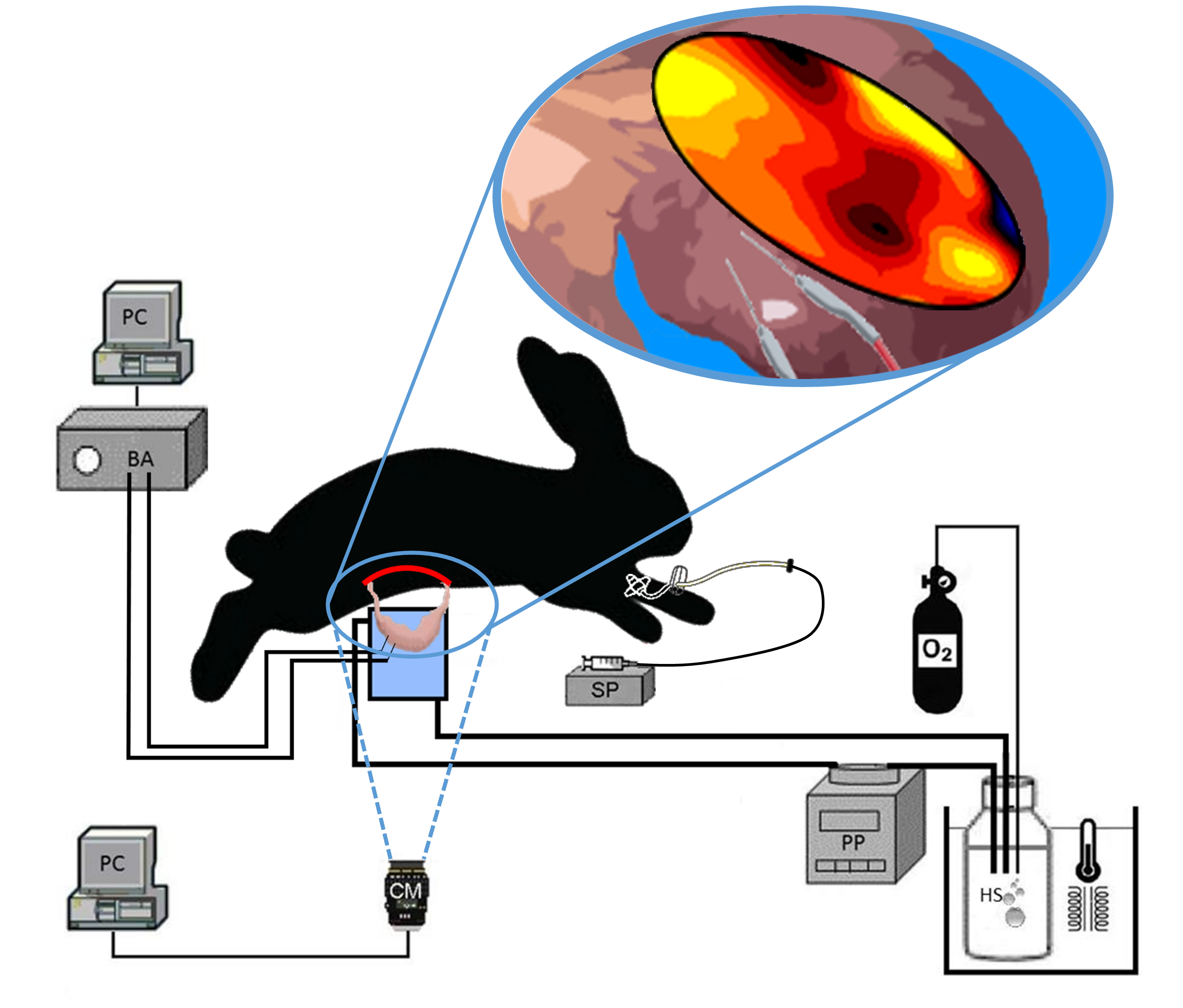

Supplement: Supplementary Figure 1 — Experimental setup for recording VSTM in the gravid uterus of the anesthetized rabbit. The anterior surface of the prolapsing uterus is positioned in the organ bath with its anterior surface to the right and filmed by an appropriately positioned video camera. The elliptical mask of the VSTM covered 45% of its anterior surface. The electrophysiological electrodes were positioned just outside of the filmed area. PC, personal computer; BA, bioamplifier; CM, video camera; SP, syringe pump; PP, peristaltic pump; HS, reservoir of Earle –Hepes solution. [file Image_1.TIF]

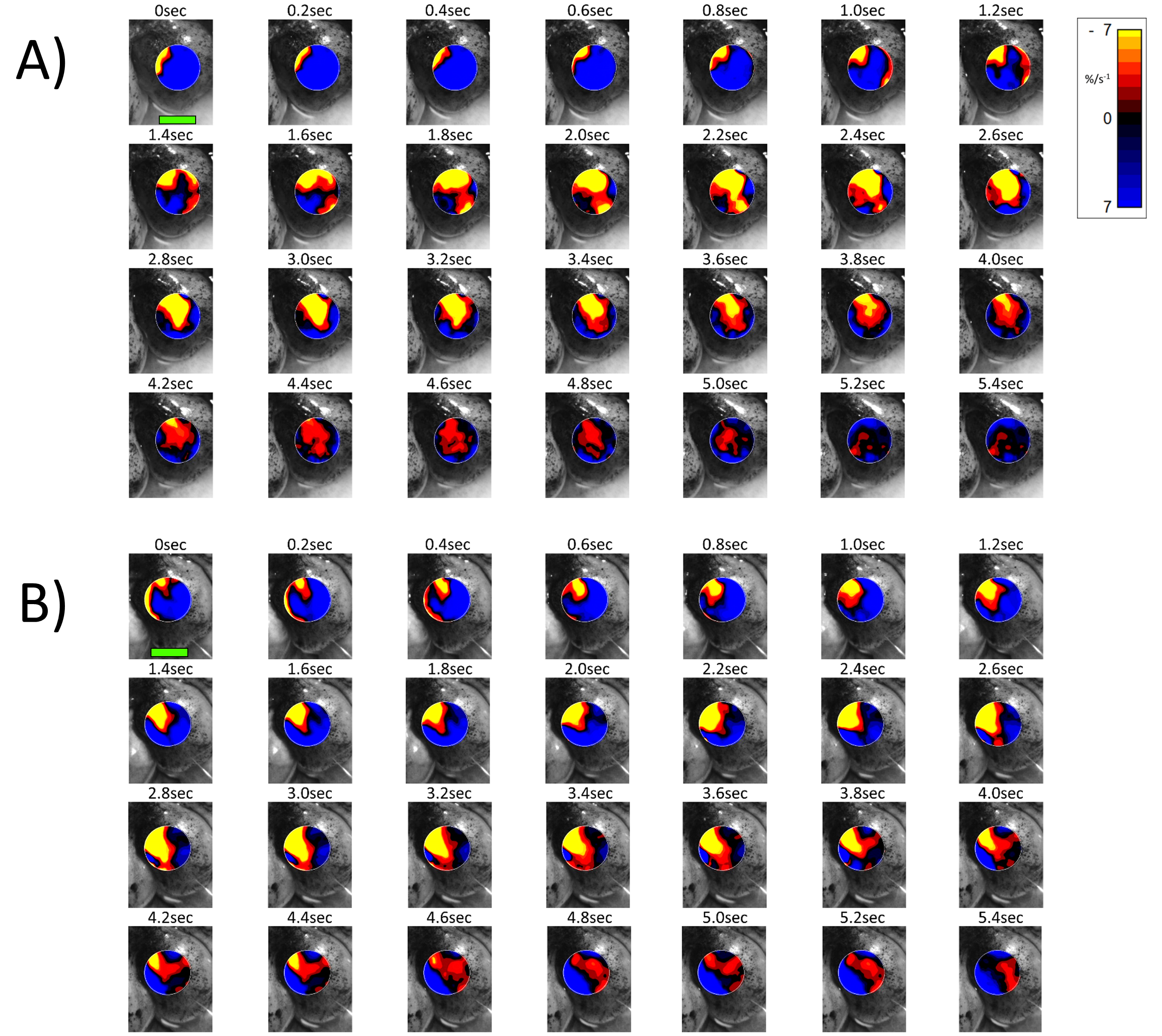

Supplement: Supplementary Figure 2 — Temporal sequence of two-dimensional maps showing variation in area strain from spontaneous contractions in a representative rabbit uterus at 20 days gestation before (A) and after (B) dosage with 16 U of oxytocin. Decrease in size from active contraction shown as red (high) and yellow (medium) levels of negative strain rate with stasis or relaxation shown in blue. Green scale bar at time 0 s in each contractile sequence represents 10 mm. [file Image_2.TIFF]

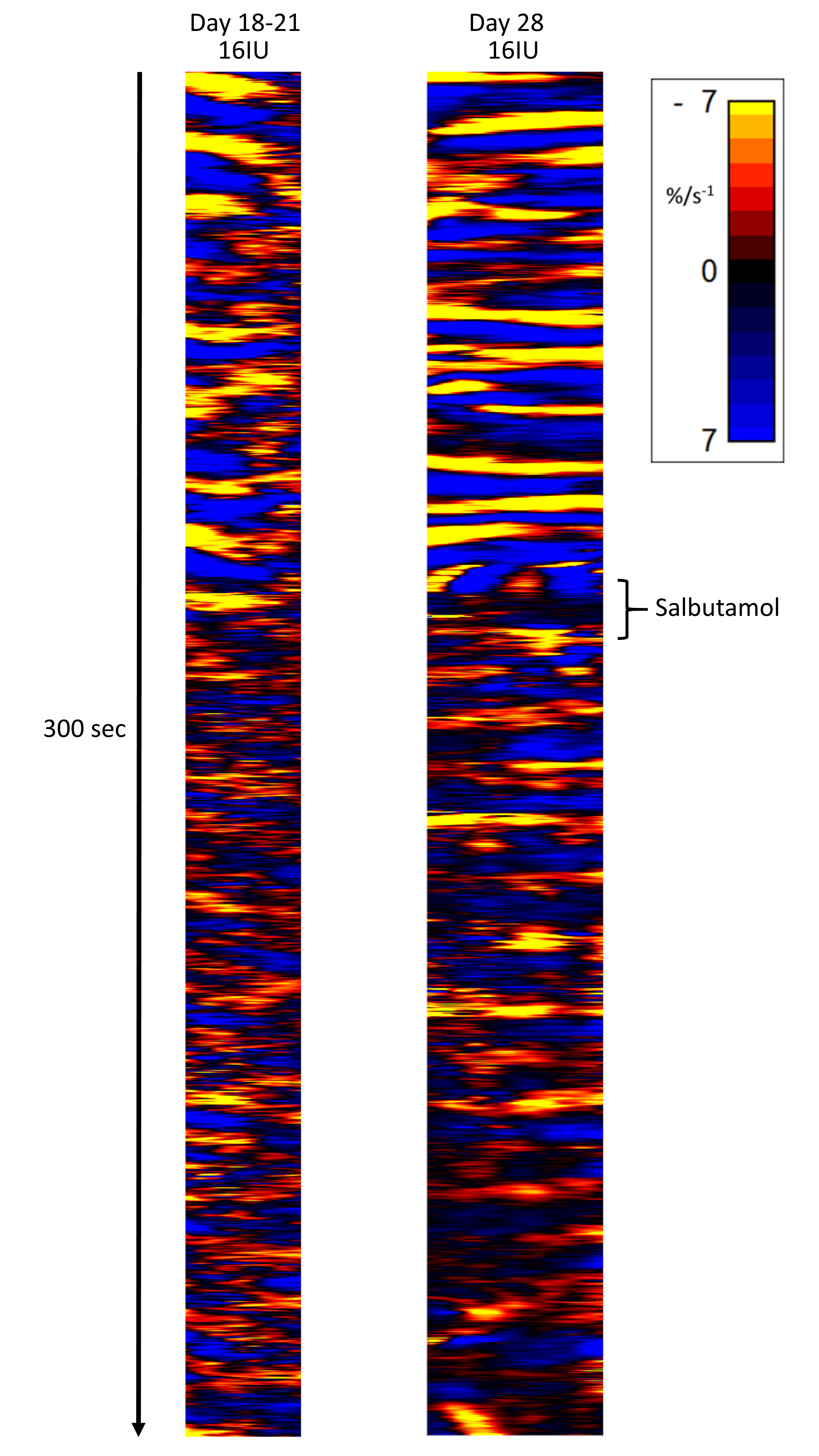

Supplement: Supplementary Figure 3 — Temporal sequence of unidimensional maps showing variation in area strain rate from component contractions after commencement of continuing maximal dosage with oxytocin a rabbit uterus at 18–21 and 28 days gestation and subsequent dosage with salbutamol (174 nmol/L). [file Image_3.TIFF]
